# A Potential Lock-Type Mechanism for Unconventional Secretion in Fungi

**DOI:** 10.3390/ijms20030460

**Published:** 2019-01-22

**Authors:** Michèle Reindl, Sebastian Hänsch, Stefanie Weidtkamp-Peters, Kerstin Schipper

**Affiliations:** 1 Institute for Microbiology, Heinrich Heine University Düsseldorf, Universitätsstraße 1, 40225 Düsseldorf, Germany; michele.reindl@hhu.de; 2 Center for Advanced Imaging, Heinrich Heine University Düsseldorf, Universitätsstraße 1, 40225 Düsseldorf, Germany; sebastian.haensch@hhu.de (S.H.); stefanie.weidtkamp-peters@hhu.de (S.W.-P.)

**Keywords:** cell separation, chitinase, fragmentation zone, fungi, septum, unconventional secretion, Ustilago maydis

## Abstract

Protein export in eukaryotes can either occur via the classical pathway traversing the endomembrane system or exploit alternative routes summarized as unconventional secretion. Besides multiple examples in higher eukaryotes, unconventional secretion has also been described for fungal proteins with diverse functions in important processes such as development or virulence. Accumulating molecular insights into the different export pathways suggest that unconventional secretion in fungal microorganisms does not follow a common scheme but has evolved multiple times independently. In this study, we review the most prominent examples with a focus on the chitinase Cts1 from the corn smut *Ustilago maydis*. Cts1 participates in cell separation during budding growth. Recent evidence indicates that the enzyme might be actively translocated into the fragmentation zone connecting dividing mother and daughter cells, where it supports cell division by the degradation of remnant chitin. Importantly, a functional fragmentation zone is prerequisite for Cts1 release. We summarize in detail what is currently known about this potential lock-type mechanism of Cts1 secretion and its connection to the complex regulation of fragmentation zone assembly and cell separation.

## 1. Introduction

Protein secretion is of extraordinary importance for living cells. The traditional view of protein export in eukaryotes encompasses the signal peptide-mediated transfer through the endomembrane system. After entering the endoplasmic reticulum (ER) and proper folding, proteins are translocated to the Golgi apparatus and then packed into secretory vesicles, which fuse with the cytoplasmic membrane to release their content into the extracellular space [1]. On their route, proteins are often subject to pathway-specific post-translational modifications like *N*-glycosylation or disulfide bond formation [2,3].

In the past years, a growing number of proteins has been described that circumvent this conventional secretory pathway and exit the cell by alternative mechanisms collectively termed unconventional secretion [4,5,6]. Most unconventionally secreted proteins are easy to identify due to the lack of the typical N-terminal signal peptide for ER entry. However, in some cases these signals are present and the proteins enter the ER, but later on the pathways divert in that the Golgi transfer is omitted (Golgi bypass) [4]. The diverse unconventional secretion pathways include both vesicular and non-vesicular export routes [7]. Importantly, the exported proteins often have fundamental cellular functions in cell survival, development, or signaling, and in many cases their secretion is strictly regulated or stress-induced [4,6,8]. Some unconventionally secreted proteins like the fibroblast growth factor 2 (FGF2) have been studied in depth and molecular details of their secretory mechanisms have been uncovered [9]. FGF2 is a cell survival factor implicated in tumor-induced angiogenesis and follows a self-sustained translocation mechanism across the plasma membrane of human cells. The process requires binding to the phosphoinositide PI(4,5)P2 at the inner membrane leaflet which results in oligomerization into colloidal membrane pores through which FGF2 monomers are released. Surface heparan sulfate proteoglycans mediate the disassembly at the outer membrane leaflet. The interaction with the α-subunit of the Na/K ATPase, ATP1A1, and phosporylation by plasma membrane-associated Tec kinase supports FGF2 export [10]. Intriguingly, recent studies indicate that membrane pore formation might also constitute the underlying principle for secretion of other relevant proteins like human immunodeficiency virus trans-activator of transcription (HIV-TAT) and interleukin 1 beta (IL-1β) [6,11,12].

While the export mechanism of FGF2 is well studied, for the vast majority of proteins the exact pathways are still largely unknown. In this review, we summarize recent findings in the field of unconventional secretion for fungal microorganisms with special emphasis on the secretory mechanism of chitinase Cts1 in the corn smut *Ustilago maydis*.

## 2. Unconventional Secretion in Fungi

Classical N-terminal signal peptides for ER entry can be predicted bioinformatically with high confidence, because they show a conserved architecture of positively charged, hydrophobic, and polar stretches with an adjacent signal peptidase cleavage site usually preceded by three small uncharged amino acids [13]. Using proteomic studies, a multitude of proteins has been detected outside the cell of fungal microorganisms despite the absence of such detectable N-terminal signal peptides. Unexpectedly, these proteins include typical cytosolic proteins like metabolic enzymes or chaperones which might fulfill moonlighting functions. Several candidates also represent virulence factors and allergens or are required for cell wall dynamics [14,15]. However, to date, unconventional secretion of only a few of these proteins has been experimentally verified. The corresponding secretion mechanisms have been studied to differing extents with representatives in both yeasts and filamentous fungi. The accumulating mechanistic details indicate that, also in the case of lower eukaryotes, unconventionally secreted proteins do not follow a common pathway but rather a diverse range of export routes that have evolved differentially. In the following sections, we summarize the best characterized examples from both yeasts and filamentous fungi and their roles in fungal physiology.

The best studied unconventionally secreted fungal protein by far is the acyl-coenzyme A (CoA) binding protein Acb1 which mediates sporulation in the two ascomycetous yeasts *Saccharomyces cerevisiae* and *Pichia pastoris* (syn. *Komagataella* spp.). Interestingly, Acb1 homologs are present in all eukaryotes, and functional conservation of the mechanism has been demonstrated not only in other fungi like the filamentous fungus *Aspergillus oryzae* and the human pathogen *Cryptococcus neoformans* [16,17], but also in the amoeba *Dictyostlium discoideum* and in mammalian cells [18]. The *D. discoideum* acyl-CoA binding protein homolog AcbA is secreted upon nutrient starvation, followed by cleavage to generate a small peptide called SDF-2, which is required for spore viability [19]. Based on this observation, a *D. discoideum* assay based on the induction of spore formation in response to AcbA homologs is widely applied as a bioassay to characterize the fungal AcbA homologs. The secretory mechanism itself is best studied in *S. cerevisiae*. Additionally, in this prominent fungal model, Acb1p is secreted during nutrient starvation [20]. Using a di-acidic motif, the protein enters structures termed compartments for unconventional protein secretion (CUPS). These compartments are composed of membranes derived from the Golgi apparatus and endosomes, and likely mediate sorting of Acb1p for export. Grh1p, a Golgi reassembly-stacking protein (GRASP) homolog known as a structural Golgi membrane protein, is essential for the entry of cytoplasmic Acb1p into the CUPS [21]. Afterward, Acb1p is packed into endosomes which are incorporated into multivesicular bodies (MVBs) by an endosomal sorting complex required for transport (ESCRT)-I-, II-, and III-dependent process. Secretion is finally mediated by fusion of the MVBs with soluble N-ethylmaleimide-sensitive factor attachment protein receptors (SNAREs) like Sso1p [22]. Of note, a similar secretory mechanism has been proposed for superoxide dismutase Sod1p from *S. cerevisiae* [18]. In humans, a mutant form of Sod1 is implicated in amyotrophic lateral sclerosis (ALS). Therefore, studies in the simple eukaryotic model organism *S. cerevisiae* might allow deciphering the impact of unconventional Sod1 secretion on ALS pathology [23].

In the filamentous fungus *Aspergillus niger*, recent evidence suggests that the aspartic protease PepN (formerly described as PepAb) [24] is exported unconventionally during carbon starvation most likely for fungal nutrition. Molecular details on the secretion machinery are currently unknown, however a connection to autophagy could be clearly excluded in this case [25]. Hence, the underlying secretory pathway appears to differ from the Acb1 machinery.

Interestingly, fungal plant pathogens comprise several unconventionally secreted proteins with virulence functions [25,26,27,28]. Plant pathogens secrete effector proteins to modulate their hosts. Some of these are transferred into plant cells to fulfill their virulence function [29,30]. The isochorismatase VdIsc1 was shown to be unconventionally secreted in *Verticillium dahliae* causing vascular wilt in a multitude of host plants. This phenomenon is conserved in oomycetes and, therefore, across the fungal border. The virulence factor is likely transferred into plant cells during infection and acts in suppressing salicylate-mediated innate immunity [28]. Since enzymes acting in hormone pathways usually locate intracellularly, unconventional secretion of VdIsc1 might be explained by a secondary adaptation to the pathogenic lifestyle of the plant pathogen. Similarly, avirulence proteins (AVR) lacking recognizable signal peptides were identified in the powdery mildew fungus *Blumeria graminis* f. sp. *hordei*. Transient expression assays in susceptible plant varieties indicate that they are delivered into the host where they might function as effectors and contribute to virulence [31]. Unfortunately, while the virulence function of the different fungal effectors is well studied, detailed molecular insights into their secretory mechanism are lacking.

Another remarkable observation has been made in the rice blast fungus *Magnaporthe oryzae*. While apoplastic effectors directed to the extracellular pathogen–host interaction zone are secreted conventionally via ER and Golgi, a non-conventional pathway has been proposed for those secreted effectors acting inside plant cells [27]. These cytoplasmic effectors play important roles during infection and are often strictly required for virulence. The unconventional secretion pathway involves accumulation of cytoplasmic effectors in so-called biotrophic interfacial complexes (BICs), which are plant-derived compartments located near the invading infectious hyphae. Like during conventional secretion, this type of unconventional export starts with signal peptide-mediated entry into the ER, but then diverges from the canonical pathway and involves the exocyst components Exo70 and Sec5 as well as the target-SNARE (t-SNARE) Sso1 [27]. Similar mechanisms with Golgi bypass have previously been described in other organisms, for example, for the mammalian cystic fibrosis transmembrane conductance regulator (CFTR) during specific cellular conditions [32].

Recently, the corn smut fungus *Ustilago maydis* has also emerged as a very interesting model to study novel types of unconventional secretion. The dimorphic Basidiomycete fungus can grow as yeast by budding, assuring its simple laboratory handling and efficient genetic manipulation [33]. Upon infection of its host plant maize, the fungus switches to filamentous growth, forming hyphae which penetrate the plant surface and proliferate in the plant tissue to finally undergo meiosis and form sexual spores [34,35]. Besides intense research conducted in the field of plant–pathogen interaction, diverse other fundamental aspects like cell and RNA biology and unfolded protein response as well as applied topics such as biomass degradation or secondary metabolite production are also currently studied in this fungal model [33,36,37,38,39,40].

With respect to unconventional secretion, two proteins following different export pathways are known to date in *U. maydis*. The peroxisomal sterol carrier protein (Scp2) acts as a virulence factor essential for efficient host infection [26]. Although lacking an N-terminal signal peptide, the protein can be detected in apoplastic fluids of infected maize plants, suggesting an extracellular function. Within the cell, it localizes to peroxisomes and this localization is a prerequisite for its virulence function. It was hypothesized that Scp2 might influence the membrane lipid composition and, therefore, the infection process [26]. Nevertheless, in this case also, an exact secretory route for its export is not yet known.

The second protein released via unconventional secretion in *U. maydis* is the chitinase Cts1. In the following paragraphs, we summarize our current knowledge about the exceptional secretory process of this enzyme.

## 3. Unconventional Secretion of Chitinase Cts1 in *Ustilago maydis*

Chitinase Cts1 functions during cell separation of yeast cells that divide by budding off daughter cells at the cell poles of mother cells [41]. Two consecutive septa are formed in this process, first on the mother- and then on the daughter-cell side. These septa seal off a small vacuolated compartment in between the two cells, termed the fragmentation zone, which is completely enclosed by both cell wall and membranes [42]. Fungal chitinases act in concert with chitin synthases to catalyze the disassembly and assembly of chitin, a structural component of the fungal cell wall, and are therefore important for the regulation of cell wall remodeling and morphology during polar growth and cytokinesis [43,44,45].

**Figure 1:**
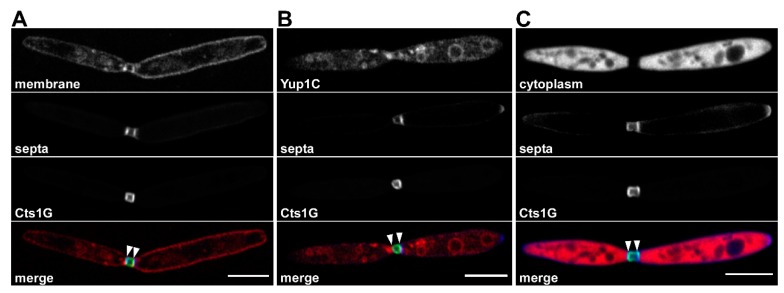
In the late stages of cytokinesis, Cts1 localizes in a small compartment connecting mother and daughter cell. Airyscan confocal micrographs of living budding cells of *Ustilago maydis*. The 32-element airyscan detector was set to super-resolution mode in order to increase resolution and signal-to-noise ratio by the subsequent reassignment of signal in the post-processing step [47]. All confocal images show budding cells prior to cell division. In all cases septa were stained with Calcofluor White (blue in merge). Cells express Cts1-eGfp (Cts1G, green in merge) accumulating in the completely assembled fragmentation zone which is sealed off by both septa and membranes (indicated by arrowheads). Scale bars correspond to 5 µm. (**A**) Fungal cells were stained with the lipophilic membrane dye FM^TM^4-64FX for visualization of the plasma membrane (red in merge). Upon prolonged incubation, the dye is endocytosed and, therefore, intracellular vesicles are also weakly stained. Cts1G is strongly enriched in the fragmentation zone. (**B**) A strain producing the early endosome marker Yup1 fused to mCherry (Yup1C, red in merge) was used. The signal can be detected in vacuoles and moving endosomes (movement not visible here) that accumulate at the fragmentation zone together with Cts1G. (**C**) A strain producing cytoplasmic mCherry was used resulting in a red fluorescent cytoplasm (red in merge). No cytoplasmic signal is visible in the completely assembled fragmentation zone.

The chitinolytic system of *U. maydis* consists of four enzymes, namely, the glycoside hydrolase 18 (GH18) family chitinases Cts1–3 and the GH20 family *N*-acetylglucosaminidase Cts4 [41]. A clear function of these enzymes during infection of *Zea mays* could not be observed, even if a quadruple deletion mutant was used [41]. In contrast, Cts1 and Cts2 function redundantly during cell separation in the yeast stage and cells lacking the two proteins form tree-like structures due to their inability to physically divide [41]. Cts1 exhibits a specific localization pattern in budding cells [41,46]. While it is found in the cytoplasm of non-dividing cells, the enzyme is translocated from the daughter-cell side to the primary septum during budding and finally accumulates in the completely assembled fragmentation zone (Figure 1) [41,46]. This localization is perfectly in line with its function during cell separation, and it was hypothesized that together with Cts2 it hydrolyses remnant chitin to break apart mother and daughter cell in the late stage of cytokinesis [41]. Similar roles during cell separation have also been proposed for chitinases in other yeasts like *Sacchromyces cerevisiae* and the human pathogen *Candida albicans* [44].

To reach the fungal cell wall, chitinolytic enzymes are typically secreted [48]. In line with this assumption, chitinases Cts2–4 harbor predicted N-terminal signal peptides, suggesting that they are exported via the endomembrane system [41,45]. In contrast, Cts1 lacks the prediction of a signal peptide. Nevertheless, yeast cells lacking *cts1* are strongly reduced in extracellular chitinase activity, suggesting that the enzyme is secreted in any case and likely follows an unconventional mechanism [49,50]. To confirm this hypothesis, a reporter system based on the bacterial enzyme β-glucuronidase (Gus) has been established [51]. Upon traversal of the endomembrane system during conventional secretion in eukaryotes, this enzyme is artificially *N*-glycosylated at a single site, leading to a strong reduction in enzyme activity [50]. Therefore, bacterial Gus cannot be secreted in an active state using the classical secretory machinery [52]. On the other hand, translational fusions of Gus to the N-terminus of Cts1 (Gus-Cts1) show activity in the culture supernatant, indicating that the ER is circumvented during this export route [50]. The N-terminal domain of about 100 amino acids is dispensable for secretion in the Gus reporter assay, further confirming the existence of an unconventional export mechanism [50].

We hypothesize that for its natural function, Cts1 sticks mainly to the fungal cell wall. This assumption is supported by its natural ability to bind chitin [53]. Detection of Gus-Cts1 in the culture supernatant indicates that this cell wall localization is (in part) blocked upon distinct translational fusions at the N-terminus, leading to the release of the fusion protein into the culture supernatant [50]. This observation is currently exploited for the establishment of a protein production platform based on unconventional Cts1 secretion (see below) [53,54].

## 4. Insights into the Mechanism of Unconventional Cts1 Secretion

**Figure 2:**
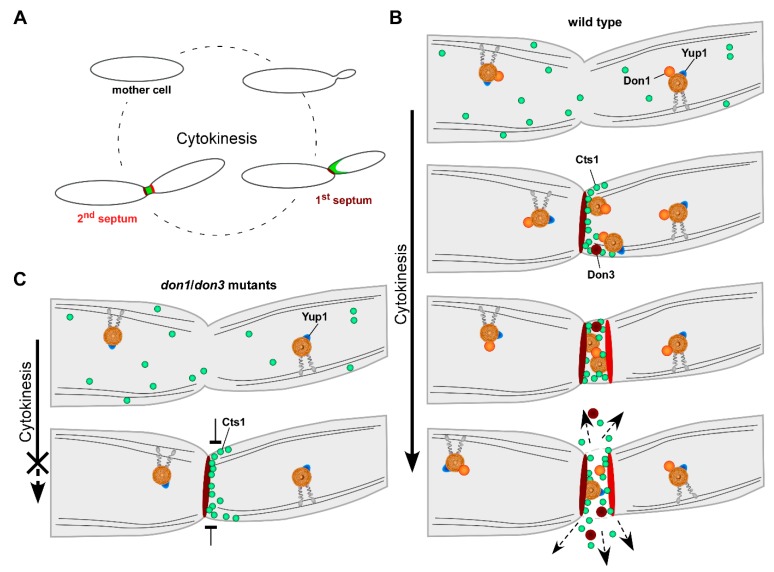
Current model of unconventional secretion via the fragmentation zone in *U. maydis*. (**A**) Cts1 (represented in green) localization is dependent on the cell cycle stage. During early cytokinesis, it is transferred to the daughter-cell side of the primary septum (dark red). As soon as the secondary septum (red) is formed, it is entrapped in the fragmentation zone from where it is released. The progress of cytokinesis is indicated by the dotted circle (clockwise). (**B**) Model of wild typic unconventional Cts1 (green circles) secretion mediated by the fragmentation zone. Primary (dark red) and secondary (red) septa are formed sequentially during cytokinesis. Early endosomes (brown vesicles) positive for the markers Yup1 (blue) move along microtubule tracks (dark grey lines) throughout the fungal cells using motor proteins (grey). Don1 (orange circles) is attached to the endosomes via a FYVE domain. During cytokinesis, Yup1-positive endosomes accumulate at the daughter-cell side of the primary septum. In addition, Don3 (dark red circles) and Cts1 are translocated to this subcellular site. Black arrows indicate the site of unconventional secretion. (**C**) Don1 and Don3 are essential for secondary septum formation and cells lacking either protein are not able to separate. Although Cts1 is still recruited to the daughter-cell side of the primary septum, it cannot be detected extracellularly, suggesting that unconventional secretion requires a functional fragmentation zone. Impeded secretion is indicated by the flat arrows. Models were adapted from Göhre et al. [57].

Its specific localization during cytokinesis indicated that unconventional secretion of Cts1 might be cell-cycle dependent and mediated by cell separation (Figure 2A). Cell cycle inhibition studies indeed confirmed that unconventional Cts1 secretion but not the signal peptide-mediated classical secretion of a hydrolytic enzyme stagnates upon cell cycle arrest [46]. Hence, Cts1 is likely secreted via the fragmentation zone during cell separation [46].

Cell separation is well described in *U. maydis*. The sequential assembly of the primary and secondary septa during fragmentation zone formation is strictly regulated. Initially, a stable septin collar develops at the mother–daughter neck, at the side where later on the respective septum is built up [55]. Next, an actomyosin ring is formed. Constriction of this ring by myosin motor activity results in invagination of the cytoplasmic membrane building up the first membrane barrier. Subsequently, the actual primary septum is formed. In the next step, the secondary septum is assembled accordingly (Figure 2A) [55,56]. However, at first glance, the two septa seem to be built up similarly, but their sequential formation is apparently regulated differentially.

Two proteins specifically needed for secondary septum formation are known—the guanine nucleotide exchange factor (GEF) Don1 and the germinal centre kinase Don3 [42]. Chemicogenetic kinase inhibition studies showed that Don3 triggers the dispersal of the septin filaments of the collar during secondary septum formation leading to the assembly of the contractile actomyosin ring [55,58]. Independently of Don3, Don1 activates Cdc42 signaling, thereby initiating secondary septum formation [56]. The small GTPase Cdc42 acts as a molecular switch and is a key regulator of cytokinesis and cell separation during budding growth [42,58,59]. In addition, a few more involved components like different septins or the septation protein Cdc15, a component of the actomysin ring, have been identified [56,60]. Nevertheless, the process of secondary septum formation is still not fully understood. For example, the connection between the regulatory processes mediated by Don3 and Cdc42 is currently not completely resolved.

Interestingly, Don1, Don3, and Cdc42 are translocated into the fragmentation zone during cytokinesis, thus resembling Cts1 localization (Figure 2B) [56,59,61]. Loss of Don1 or Don3 results in strong cytokinesis defects which can be attributed the mutants’ inability to form secondary septa (Figure 2C). As a consequence, cells are unable to separate physically and aggregate in tree-like structures. Due to this defect, the mutants form donut-shaped colonies on solid plates determining the protein names (donut proteins) [42]. Remarkably, both deletion mutants also show a significant reduction of Cts1 secretion. This strongly supports the hypothesis of Cts1 secretion via the fragmentation zone in a mechanism that can be compared to a lock, which regulates the transfer of boats between different river compartments [46] (Figure 2B,C). It further suggests that cell separation is a prerequisite for Cts1 release. However, we found that additional mutants displaying cell separation defects caused by rather pleiotropic effects are not impaired in Cts1 secretion. Hence, it can be hypothesized that it is not the event of cell separation itself which determines unconventional secretion but rather the accurate and functional assembly of the fragmentation zone [46].

## 5. The Fragmentation Zone as a Potential Novel Site of Lock-Type Protein Exit

With its complex regulation in space and time, the fragmentation zone is a reasonable novel compartment mediating the unconventional secretion of chitinase Cts1 in a lock-type manner. To our knowledge, no similar secretory mechanism has been described to this date in the literature. An important open question is if the fragmentation zone could not only mediate Cts1 secretion but also deal as a general site for the release of proteins or even other cellular components like the accumulated vesicles (Figure 2B). One could even speculate that the compartment is used to efficiently remove cellular garbage. Applying the established Gus reporter system to investigate unconventional secretion, we detected Don3 but not Don1 in significant amounts in the culture supernatant [46]. Extracellular functions of kinases have been described earlier, suggesting that Don3 could indeed display additional functions after secretion. However, compared to Cts1 secretion, extracellular Gus activity in Gus-Don3 expressing strains was rather low [46]. Don1 is fixed to membrane vesicles via a FYVE domain (see below) and relies on its partner Cdc42. In this case, secretion would probably not provide any further advantages to the cell. The observations support rather a model in which only distinct proteins are released, whereas others are retained in the fragmentation zone. In this case, lock-type unconventional secretion would constitute a tightly regulated mechanism.

A second point that remains unclear is how Cts1 is selected for secretion via this pathway, and what determines its specific accumulation at this site. Truncation studies have not yet deciphered a clear motif essential for Cts1 secretion or localization (unpublished observations). In contrast, some information has been gathered for the other proteins known to localize in the fragmentation zone. Interestingly, Don1 is associated with endosomal vesicles which shuttle bidirectionally along microtubules and finally accumulate in the fragmentation zone [59]. In line with this, Don1 contains a FYVE domain that specifically binds phosphoinositol-3 phosphate, an identity determinant of early endosomes. These endosomes are positive for Rab5a and harbor the endocytic marker Yup1 (Figure 1B and Figure 2B). They have been described as vesicular multi-purpose carriers which fulfill distinct functions in yeast-like and hyphal cells. In budding cells, in addition to their role in cytokinesis, they are implicated in pheromone signaling [57]. In hyphae, they mediate the long-distance transport of large mRNA subsets which guarantees unidirectional polar growth [36,62] and are involved in peroxisome distribution [57,63,64]. On the other hand, the Don1 target Cdc42 is prenylated at its C-terminus and therefore primarily localizes to the cytoplasmic membrane. The exact location of the interaction between the small GTPase Cdc42 and its GEF is currently unknown, but evidence suggests that it may also happen at the Don1-containing endosomes [59].

Apparently, Don3 also does not associate with moving endosomes. A so-called T-motif has been identified at its extreme C-terminus which is necessary and sufficient to target the protein to the fragmentation zone [61]. This motif is not visibly conserved in Cts1, and endosomal shuttling has also not been observed (unpublished). Nevertheless, it is conceivable that, similar to other organelles like mitochondrial or peroxisomes, distinct (conserved) targeting peptides exist that direct proteins into the fragmentation zone [65,66].

An additional fascinating aspect connected to secretion via the fragmentation zone arose with the recent finding that the conventionally secreted dual function metalloprotease Fly1 was implicated in the post-transcriptional regulation of Cts1 activity [67]. Together with the astonishing finding that conventionally secreted Cts2 acts redundantly with Cts1 in cell separation despite their different secretion modes [41], this adds a novel level of complexity to the yet unsolved secretory mechanism via the fragmentation zone. In both cases, it is completely unknown if and how the conventionally secreted proteins enter the fragmentation zone. It is conceivable that septum-directed secretion also occurs during signal peptide-mediated canonical secretion. Similar observations have been described for α-amylase AmyB in the septated filamentous fungi *Aspergillus oryzae* which accumulates at the septal periplasm between fungal cell wall and plasma membrane [68]. In the future, this phenomenon needs to be addressed in more detail to resolve potential interconnections between the two secretory pathways in fungi that divide by septum formation.

## 6. Conservation of Unconventional Secretion via the Fragmentation Zone

Chitinases as well as chitin synthases play an important role for the interplay between cell wall synthesis, remodeling, and degradation during growth and cytokinesis in all fungi and especially in yeasts that divide by budding [45,69]. This raises the apparent question of evolutionary conservation of Cts1 secretion. Interestingly, parallels exist between the chitinolytic system of *U. maydis* and the Ascomycete model yeast *S. cerevisiae*. Three chitinases (Cts1–3p) have been described in *S. cerevisiae* and, interestingly, Cts2p also lacks an N-terminal signal peptide [41]. At first glance, this suggests that the unconventional secretion mechanism could be conserved in budding yeasts across the borders of Basidio- and Ascomycetes. However, there are also obvious differences. A role during spore formation has been described for the Cts1 homolog ScCts2p, whereas a contribution of the enzyme to cell separation has not yet been detected [70]. Classically secreted ScCts1p is on the other hand important for cell separation in budding yeasts [71]. A ScCts1p/Cts2p double deletion mutant might probably reveal if ScCts2p is also involved in cell separation.

With respect to the secretory process, both septation factors important for unconventional secretion of Cts1 in *U. maydis*, Don3 and Don1, are not conserved in *S. cerevisiae*. This is in line with the fact that the process of septation and cell separation is clearly different from *U. maydis* and especially that no obvious fragmentation zone exists in *S. cerevisiae* [72]. This supports the hypothesis that the overall strategy of unconventional chitinase secretion might be similar between the two organisms, but the exact export mechanisms might differ. Further evidence for this hypothesis arises from additional knowledge gained about *S. cerevisiae*. Here, the zinc-finger transcription factor Ace2p specifically localizes to the daughter cell nucleus controlling the daughter cell-specific expression of proteins including ScCts1p which are required for septum assembly and cell separation [71,73]. The Mob2p-Cbk1p kinase complex regulates the activation and accumulation of Ace2p in the daughter cell nucleus. We also observed an accumulation of Cts1 at the primary septum that is elevated at the daughter-cell side. However, preliminary experiments reflecting studies performed in *S. cerevisiae* [73] did not confirm a daughter cell-specific expression of Cts1 in yeast cells (unpublished observation). This suggests that the two fungi use different regulatory circuits. In line with this, a homolog of Ace2p cannot be detected in *U. maydis*. In contrast, at least one candidate for the Cbk1 transcription factor may be present, but has not been studied so far. Hence, in summary, there is accumulating evidence that the general strategy of unconventional secretion via the fragmentation zone could be conserved in other yeasts, but the mechanistic details seem to vary.

## 7. Conclusions and Perspectives

In the past years, an increasing number of examples for the presence of unconventional secretion mechanisms in fungi has been collected. Similar to those in higher eukaryotes, these export mechanisms seem to differ substantially between fungal organisms, suggesting independent evolutionary origins. Due to the fact that these mechanisms are hard to discriminate from cell lysis, it is conceivable that we are currently misinterpreting other relevant examples. Future studies will therefore reveal the extent and impact of unconventional secretion for fungal biology and especially during virulence. Importantly, for those mechanisms that are conserved in higher eukaryotes, fungal microorganisms can serve as simple genetic models and may thus support our understanding of associated human diseases [18,74].

Substantial progress has been made towards elucidating the mechanism of unconventional Cts1 secretion in *U. maydis*. However, while basic features of the pathway have been revealed, detailed questions remain to be resolved. These include the mechanism of translocation to the fragmentation zone, the identification of proteins that not only regulate but actually act in the pathway, and a detailed view of the fragmentation zone architecture and its protein and lipid components. The question of whether the observed lock-type Cts1 secretion mechanism involves vesicular structures such as early endosomes accumulating in the fragmentation zone remains to be investigated in detail. Recent advancements concerning the mechanism of unconventional Cts1 secretion suggest that it does not fit into any of the proposed models [4] but rather constitutes a novel strategy for protein export.

Notably, the discovery of unconventional Cts1 secretion has also inspired the exploitation of this novel protein export mechanism [50,53,54]. In those studies, Cts1 is used as a carrier for the secretion of heterologous proteins. Since Cts1 circumvents the endomembrane system, this provides the unique possibility of producing heterologous proteins without *N*-glycosylation. In addition, no apparent size limitation for the secreted products exists [50]. The system has already been successfully applied for the production of reporter enzymes like the bacterial β-glucuronidase Gus and valuable pharmaceutical proteins such as antibody fragments, including single-chain variable fragments or nanobodies [50,53,54]. Additional enzyme targets are in the pipeline (unpublished). In spite of different attempts at optimization which have already led to strong improvements in the system, current yields are not yet competitive, with values at approximately 0.5 mg/L depending on the produced protein [54,75]. Importantly, insights into the molecular details of the secretory mechanism will also boost its application as a protein production platform.

## Abbreviations

BiC	biotrophic interfacial complexes
CUPS	compartments for unconventional protein secretion
eGfp	enhanced green fluorescent protein
ER	endoplasmic reticulum
ESCRT	endosomal sorting complex required for transport
GEF	guanine nucleotide exchange factor
GRASP	Golgi reassembly-stacking protein
MVB	multivesicular body
t-SNARE	target-soluble N-ethylmaleimide-sensitive factor attachment protein receptor
